# See-through display based on commercial photopolymer: Optimization and shrinkage effects

**DOI:** 10.1016/j.heliyon.2023.e16646

**Published:** 2023-05-24

**Authors:** Joan Josep Sirvent-Verdú, Juan Carlos Bravo, Jaume Colomina-Martínez, Cristian Neipp, Daniel Puerto, Andrés Márquez, Sergi Gallego

**Affiliations:** Instituto Universitario de Física Aplicada a Las Ciencias y Las Tecnologías, Universidad de Alicante. Apartado 99, 03080 Alicante, Spain

## Abstract

Abstract

Nowadays augmented reality, 3D Image, mixed reality and see-through applications are very attractive technologies due to their great potential. Holographic optical elements can provide interesting solutions for injection and extraction of the image in the waveguides that are part of the see-through devices. We have developed a coupled waveguide system based on slanted transmission gratings recorded in manufactured photopolymers. In this work we optimize our schedule to a commercial photopolymer for this high demanded application. We demonstrate that high diffraction efficiencies can be obtained if we optimize the recording geometry, recording intensity and recording time for this material. In addition, we study the effects of shrinkage in our holographic system. In general shrinkage is an important drawback for holographic applications, nevertheless we demonstrate how shrinkage can help these systems open new possibilities. Lastly, we show how to significantly improve the quality of the guided image.

## 1 Introduction

Augmented reality (AR) has become one of the more important hot topics in the field of optics. This technique lets the users observe virtual computer-generated perceptions in real-world environments [[1], [2], [3], [4], [5], [6], [7], [8], [9], [10], [11]]. Nevertheless, the work to obtain an appealing commercial product is still on the table. This technology can be used on smart glasses or on the car industry, front glasses; nonetheless, some drawbacks regarding the resolution, the field of view and the power consumption must be overcome., [1]. There are many steps involved in the see-through process that should be fixed, such as the input and output elements, retinal image projection, the tunability, the propagation for curved waveguides, etc.

One of the most promising solutions for the in-coupler and out-coupler elements fabrication are the holographic optical elements [1,12]. Most of the recording holographic geometries are based on reflection holograms using prisms, and microlens arrays [6]. The reflection geometry has the main difficulty that the holographic recording material needs high spatial resolution and more stability in the lab, because the spatial resolution required for the material is over 4000 lines/mm. In previous papers we proposed an alternative geometry to record these elements based on transmission gratings [11,12]. The main idea of this method is the fabrication of holographic recording elements using a shorter wavelength than the selected to be guided in the see-through display. We optimized the best transmission recording geometry for three different photopolymers prepared in our lab [11]: polyvinyl-acrylamide based photopolymer [[12], [13], [14], [15]], PVA/AA, with a functionality slightly higher than one, a nanoparticle-(thiol-ene) polymer composite dispersed with SiO_2_ nanoparticles [[16], [17], [18]] and average functionality higher than two, NPC, and a holographic photopolymer with dispersed liquid crystal molecules (HPDLC) [19,20], with average functionality higher than five.

Photopolymers are ideal candidates as a recording holographic material for many applications [13] and, to fabricate in-couplers and out-couplers. They present low scattering, have low cost, and high diffraction efficiencies, furthermore, we can fabricate tunable holograms with electric [20,21] or magnetic fields [22]. Nevertheless, the preparation of the photopolymer requires high precision and control of the laboratory’s conditions. In this sense, commercial photopolymers such as Bayfol [[23], [24], [25], [26]] exhibits high repeatability and easy management, therefore is an appealing solution for users not experts dealing with holographic recording materials. This commercial photopolymer presents a good response for transmission and reflection gratings as was characterized in these ref. [[23], [24], [25], [26]]. In this work we analyze and optimize our holographic technique to fabricate in and out-couplers for see-through application for Bayfol HX 200.

## 2 Theoretical background and experiments

In the regime of volume holography only two diffracted orders are significant, for which happens to be an energy-coupling effect due to the diffraction grating. Given the wavevector of both the incident and the diffracted beam, ρ and σ respectively, the grating vector **K** can be computed as follows:

(1) K=ρ−σ

where |K|=2π/Λ and |ρ|=|σ|=2πn/λ, Λ is the grating period, λ is the wavelength and n is the average refractive index of the sample (for Bayfol HX 200, n = 1.505).

**Fig. 1:**
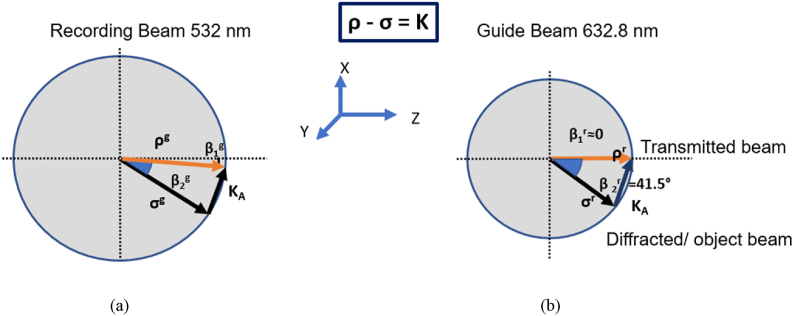
Ewald’s sphere for Geometry A: recording with green light (a) and read out in the Bragg condition, designed for normal incidence with red light (b).

The recording and read out schemes inside the material to guide red light with normal incidence are represented in Fig. 1 using Ewald’s sphere, where **K** is the grating vector that can be obtained easily from (1) given the two interfering wave vectors, ρ and σ**.**

Moreover, to assure the total internal reflection that allows the grating to be a coupler in a waveguide, it must be accomplished that the diffracted beam is deflected so that its propagation angle with respect to the normal of the glass substrate is higher than the critical angle $\theta_{c}$. Hence, as the sample is affixed to a glass with 2 mm of thickness, the different geometries presented in this work seek to enable the waveguide function.

In previous works we have shown how depending on the recording geometry the materials present different behavior and diffraction efficiency. There is only one configuration to obtain guided light with normal incidence, geometry A (Table 1), but if the incident angle is flexible, then we can change two parameters, the tilted angle of the hologram fringes or the spatial frequency of the grating. There are materials that support high spatial frequencies and others high slanted gratings values. In this sense, we must check which geometry is optimum for Bayfol photopolymer. In Table 1 the wave vectors are given inside the material and outside, in air, for the two wavelengths used. For example, $\sigma_{g}$ and $\sigma_{r}$ represent the vectors for green and red respectively.

**Table 1:** Values of the recording wave and grating vectors with the different geometries used. Also, the theoretical values for the guided 632.8 nm light.

Geometry	Recording angle 1 in air $\beta_{1}^{g}$ (°)	Recording angle 2 in air $\beta_{2}^{g}$ (°)	Modulus K (μm^−1^)	Slant angle φ (°)	Bragg angle in air $\beta_{1}^{r}$ (°)	Diffracted angle inside the material $\beta_{2}^{r}$ (°)
A	−4.8	−69.2	10.8	69.13	−0.46	−42.05
B	4.8	−69.2	12.6	72.32	−11.15	- 42.76
C	−29.9	−74.0	6.3	60.38	−26.70	−41.81
D	−27.8	−69.2	6.3	61.67	−24.66	−40.52

As we mentioned before one of the most interesting geometries for the couplers is geometry A, where the incident beam is guided through the waveguide with normal incidence and the out beam to the eye is also normal to the glass. Nevertheless, more geometries can be proposed to improve the diffraction efficiency of the photopolymer, looking for less slanted fringes or smaller spatial frequencies. That is why we propose geometry B, with less tilted angles of the fringes and higher spatial frequency and geometry C with higher tilted angle and smaller spatial frequency.

**Fig. 2:**
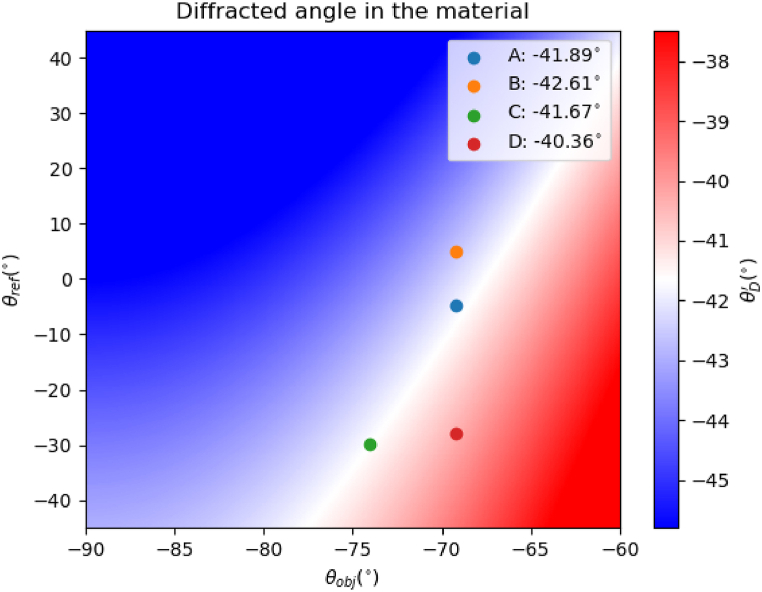
Propagation angles of the object and reference beams compared with the critical angle. Blue color means that the diffracted beam is guided, and red color means that theoretically the diffracted beam escapes.

To have a deeper insight into the geometries recorded and the angles of the red propagation inside the material we have presented Fig. 2. In this figure, the angle of the diffracted beam is represented as a function of the object beam and reference beam angle propagation inside the material during the recording process, where the limit angle to obtain total reflection with 632 nm is depicted with white color. It is important to remark that geometry D is not inside the blue region, therefore theoretically light would escape from the glass. Due to the changes in the grating's planes in the material, fringes space, and inclination during recording this geometry can be also used to fabricate these see-through displays.

In Table 1 we have shown the recording angles in air with a wavelength of 532 nm and the value of the modulus of K and its tilted angle to the horizontal for each geometry. The theoretical Bragg angle in air for 632.8 nm is also included, which is the angle between the transmitted beam and the z-axis in Fig. 1.

It is important to note that, due to shrinkage, these Bragg angles experimentally change. In this sense this detuning can be used for estimating the shrinkage values for each geometry. Due to the shrinkage, usually the slant angle of the grating also changes [[27], [28], [29]], this effect can produce guided light, total internal reflection inside the glass, even for geometries where theoretically it is not expected. To prove this phenomenon, we have also recorded the geometry D. For this geometry the expected angle in air for the diffracted order is 77.0° and 40.5° inside the material.

**Fig. 3:**
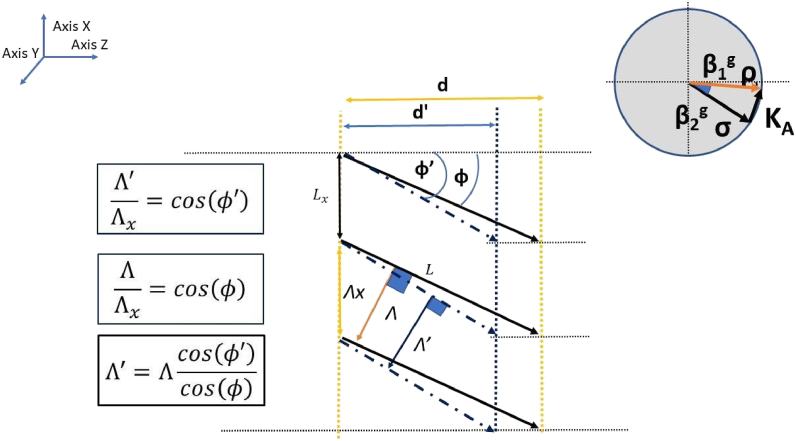
Model of shrinkage proposed in Ref. [27]. Light travels in the positive direction of axis Z. The Ewald’s sphere.

In general, considering some approximations we can model the shrinking process for slanted gratings as in Ref. [27]. In this model it is assumed that the x component of the period remains constant and there are only changes in the z component (see Fig. 2). Taking this assumption into consideration, it can be deduced the relation between thickness changes, the spatial period of the grating, and the initial tilted angle as follows:

(2) $$\frac{\Delta \Lambda }{\Lambda }={\left(-\cos\left(\varphi \right)\right)}^{2}\frac{\Delta d}{d}$$

where d is the thickness, Δd is the shrinkage, Λ the spatial period, φ the angle of the fringes with the horizontal and ΔΛ is the variation of the period, as is depicted in Fig. 3. In this figure the recorded fringes are represented by continuous lines and separated by a spatial period Λ. The fringes after shrinkage are represented by discontinuous lines and separated by a new period $\Lambda^{\prime }$, with a new thickness $d^{\prime }$. L is the maximum length of the fringes [27] and $\Lambda_{x}$ and $L_{x}$ are two constants independent of the shrinkage and derived from the assumed conservation of the x component of K, $K_{x}$ in this model.

The initial period is related to the interference beams angles by (1) and there are two ways to obtain the period after the holographic grating recording: either if the component $K_{x}$ remains constant and measuring only the +1 Bragg’s angle or considering +1 and −1 diffracted order angles and re-calculating the whole grating vector. An alternative method consists in obtaining the period from the Bragg’s condition; in general, Bragg’s condition can be obtained from the next equation:

(3) $$\frac{2n}{\lambda }\sin\left(\theta \right)=\frac{1}{\Lambda }$$

where θ is the angle between the replay vector and the fringes. As usually after shrinkage it is difficult to precise the slanted angles of the fringes, it is possible to use an alternative way where Bragg’s condition is expressed as a function of the angle formed by ρ and σ inside the material, φ, as follows:

(4) $$\frac{2n}{\lambda }\sin\left(\varphi /2\right)=\frac{1}{\Lambda }$$

and here we must measure the two Bragg’s angles, corresponding to +1 and −1 diffracted orders. With this method the error in the determination of the shrinkage is drastically reduced [27].

It is important to note that the −1 diffracted order in the case of waveguide couplers cannot be measured by guided light, 632.8 nm in this case, then to use this method we must determine the Bragg’s angles with the recording wavelength, 532 nm, which haven’t total reflection. We have followed this method of calculation to reduce the error, for which (3) and (4) are obviously still valid.

The holographic experimental set-up is like the presented in previous papers, nevertheless due to the broad absorption of the Bayfol HX 200 it is impossible to read the hologram formation in real time using red light. Therefore, we have recorded many holograms using different exposure times and after the curing process, the angular response of the recorded hologram is measured using a He–Ne laser for each one and with 532 nm to calculate the shrinkage using (2).

## 3 Results and discussion

### 3.1 Geometry for normal incidence: time and intensity optimization

Due to the high asymmetry of the holographic recording the intensities of the two arms must be adjusted to obtain an intensity ratio at the material close to 1:1 to maximize the fringes visibility like follows:

(5) $$I_{\sigma }\;\cos\left(\theta_{\sigma }\right)=I_{\rho }\;\cos\left(\theta_{\rho }\right)$$

Therefore, for Geometry A the relation $I_{\sigma }/I_{\rho }$ must be around 2.8. We have checked both different recording intensities and exposures times using 532 nm.

In a recent publication the saturation [26] regime was achieved with a dose of 20 mJ/cm^2^ for a recording wavelength of 532 nm for transmission gratings with spatial frequencies around 1200 lines/mm. We have tested this commercial photopolymer under different recording intensities for geometry A and different exposures, all inside the saturation regime and we expect similar diffraction efficiencies (DE).

The DE is obtained from the values of the transmission efficiency (TE), as the diffracted light cannot be directly measured because it is guided inside the waveguide. Hereon, we present the results regarding the relative efficiency (6), where Fresnel and absorption losses are not considered. $I_{0}$ holds for the zeroth order intensity and $I_{t}$ for the transmitted intensity from the exposed material when there is no hologram:

(6) $$DE=1-\frac{I_{0}}{I_{t}}$$

**Fig. 4:**
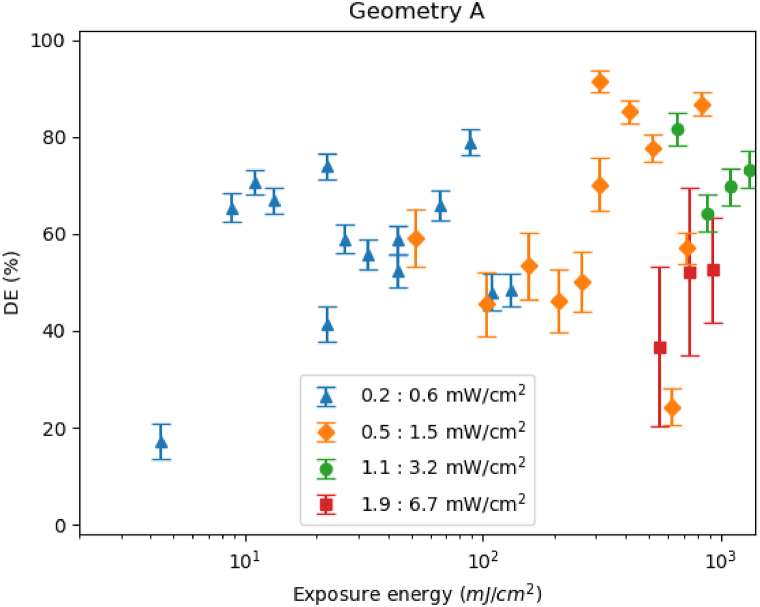
Diffraction efficiencies obtained using different recording intensities for geometry A.

The measured values of the DE as a function of the exposure energy are presented in Fig. 4, which are good enough for every case and are like those obtained with HPDLC in previous works [11] (in that case the couplers can be also tunable [20]). It is important to remark that the repeatability of the Bayfol is much higher than the HPDLC manufactured photopolymers. In general, the material presents good behavior for this recording geometry and the average guided intensity along the glass is 70% of the possible. The point with less than 20% of DE is due to the low energy exposure (only 5 mJ/cm^2^), not reaching the saturation regime.

**Fig. 5:**
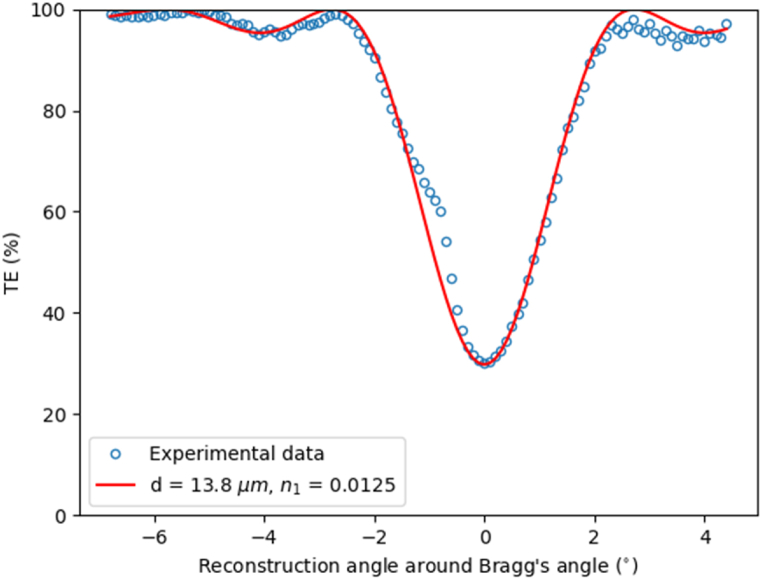
Angular response of a hologram for geometry A, and data obtained fitting the experimental data.

The best results obtained with low intensities agree well with previous studies and it is due to the more importance of the diffusion process when the polymerization is slower. Once the sample is cured, we measured the angular response of the hologram, and we fitted the experimental data using Kogelnik theory for slanted gratings [30], Fig. 5. From this fitting we extract the optical parameters of the grating: its refractive index modulation $n_{1}$, and the effective optical thickness d. In every case the effective optical thickness [31] is between 13 and 15 μm and the refractive index modulation between 0.010 and 0.016. It is worth noting that the optical thickness of the layer is smaller than the physical one due to the light absorption of the photopolymer. In the case of transmission gratings both beams impinge on the same side of material, the transmittance of the materials for 532 nm is around 45%, then the grating formation in the back side of materials is weaker than the front side.

**Fig. 6:**
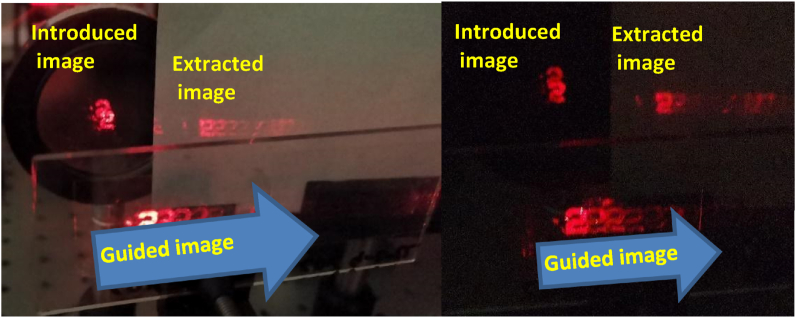
See-through system working with in and out-couplers fabricated using Geometry A and Bayfol HX 200 as photopolymer. With ambient light and in dark.

In Fig. 6 we have represented the guided image through our system with in and out holographic couplers fabricated with geometry A. The image of a number 2, which size is 1 cm, using an optical test target illuminated by a collimated He–Ne beam is introduced in our system, that can guide it and extract it successfully. The input and output couplers are circles with 3 cm radius and the area of the holograms can be controlled using a mask during the recording process.

Once the image is guided and extracted from the display it is projected on a white screen. We obtain some ghost images but with clearly less energy than the first one [32]. The ghost images are caused by different reasons. The diffraction efficiencies do not reach the 100% (85% in the system presented in Fig. 6) and the Fresnel reflections are high for tilted angles and refractive index variations from 1.505 to 1. Besides, due to the big size of the hologram, 3 cm diameter, compared to the image, 1 cm diameter, there is a short distance between two consecutive reflections inside the glass substrate, that superimpose the successive generated images. The results obtained by geometries C or D are quite similar, only changing the input and output angles.

As we have not used lenses in this system and the beam is collimated, it makes no sense to measure the field of view (FOV) [33], as the maximum size of the guided image corresponds with the size of couplers, 3 cm of diameter.

Analyzing the results presented in this section, the best and most consistent results are obtained using the recording intensities for each arm of 0.5:1.5 mW/cm2, for which each of the gratings has a DE higher than 80% (Fig. 4). Considering the angles of geometry A, the total intensity received by the sample is around 1 mW/cm2. Then for the alternative geometries we use this value as a reference, which will provide different exposure energies, as we choose to vary the exposure time between samples.

### 3.2 Different recording geometries

**Fig. 7:**
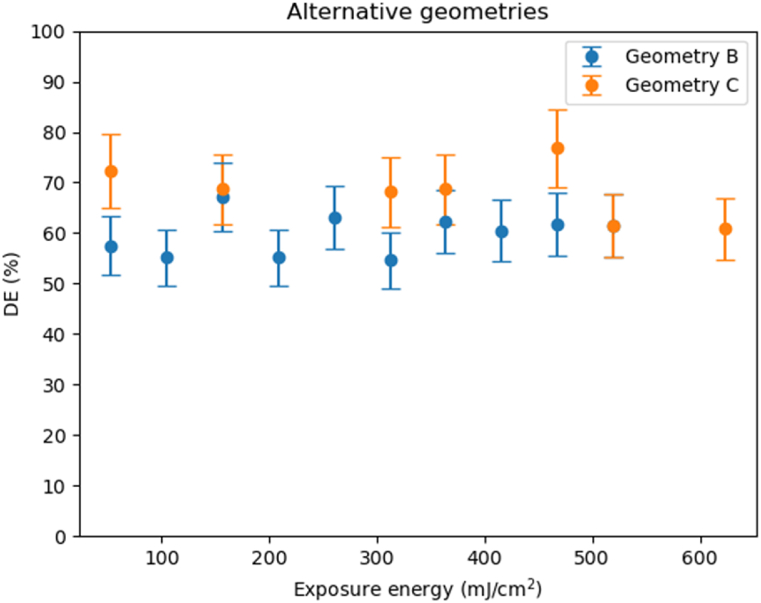
Diffraction efficiencies obtained using different exposure energies for geometry B. and for geometry C. Intensity ratio of the beams were 0.5: 1.5 mW/cm^2^, for geometry B and 0.7:2.0 mW/cm^2^ for geometry C.

As we demonstrated in previous works each photopolymer has an optimum recording schedule for the recording process of the in and out holographic couplers. There are materials based in PVA/AA that have problems with very slanted gratings due to the low value of the polymer functionality [11] and the high value of the shrinkage. Others, such as NPC, present more problems related to high spatial frequencies due to the long length of the polymer chains [23]. To optimize the holographic recording scheme for Bayfol HX 200, we have checked the material behavior under geometry B (higher spatial frequency and less slanted) and C (low spatial frequency and more slanted), and we have compared the obtained results (Fig. 7) with the ones with geometry A. The beam intensities were selected to be close to the optimized in our lab conditions, achieving 0.5:0.5 mW/cm^2^ in Eq. (5).

### 3.3 Shrinkage effects

For many applications, such as holographic data storage, shrinkage is one the main drawbacks of photopolymers materials, but it can become an advantage for see-through applications using our proposed technique.

Due to the shrinkage the spatial period can increase, and the slanted angle of the fringes also (the angle of the vector **K** reduces). It is possible for the customers to read the value of the shrinkage for Bayfol HX 200 at the section of “Description and applications”, where the shrinkage for reflection gratings presented is 1.4%. In the case of our geometry the spatial frequencies are clearly lower than the reflection ones.

To analyze the obtained results, we present Table 2. In previous papers we have shown that to improve the precision of the shrinkage measurements it is important to consider the detuning of one of the higher diffracted orders [12,27]. The real values of the vectors **ρ** and **σ** were measured with the recording wavelength, in opposition to Ref. [27] where a He–Ne laser was used, because in this case one of the angles in air would be higher than 90° for 632.8 nm wavelength. Both parameters of recording grating vector K are important to know whether the red light is guided or not, in other words, if the angle inside the material is higher than 41.64° that corresponds to the total reflection for exposed photopolymer of Bayfol HX 200 which is n = 1.505.

**Table 2:** Interval shrinkage for each recording geometry, angle of K vector and the angle of order +1 inside the material.

Geometry	Shrinkage (%)	Slanted angle (°)	$\beta_{2}^{r}$ angle inside (°)
A	0.2–1.5	69.1–69.3	>41.8
B	1.0–4.0	72.0–72.5	>42.5
C	0.6–3.1	60.2–60.6	>42
D	1.3–3.3	60.7–62.4	40.9–41.8

From the data presented in Table 2 we can see how the shrinkage helps our proposed schemes to guide the light in the substrate. That means that we have more flexibility to design the recording schemes to generate these in and out-couplers. Other alternative recording schemes were recently presented in Ref. [33], but the shrinkage and the variation in the tilt of the grating planes were not investigated.

**Fig. 8:**
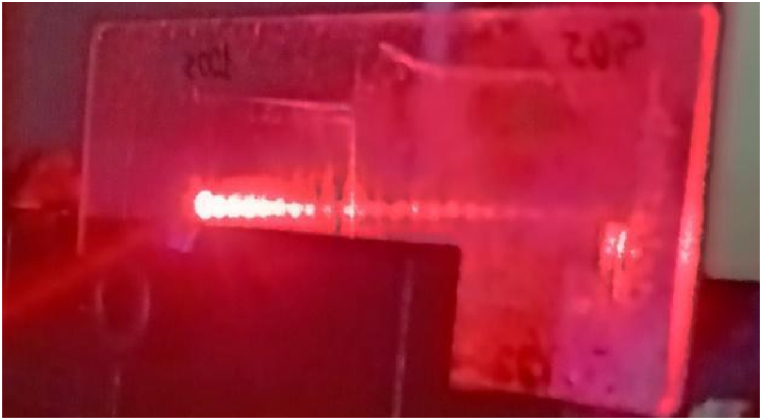
Light guided using geometry D with diffraction efficiency of 76%.

For geometry D we are just at the limit and sometimes the light is not guided. We have checked this geometry with other materials with more shrinkage as PVA/AA materials and for them the light is always guided using geometry D, for this material the shrinkage is higher than 4% for this geometry D. We present in Fig. 8 the holographic waveguide fabricated with this recording configuration, where theoretically the light cannot be trapped, but we have checked that the design is able to accomplish the desired task, to guide the light along the substrate, as the other geometries do.

**Fig. 9:**
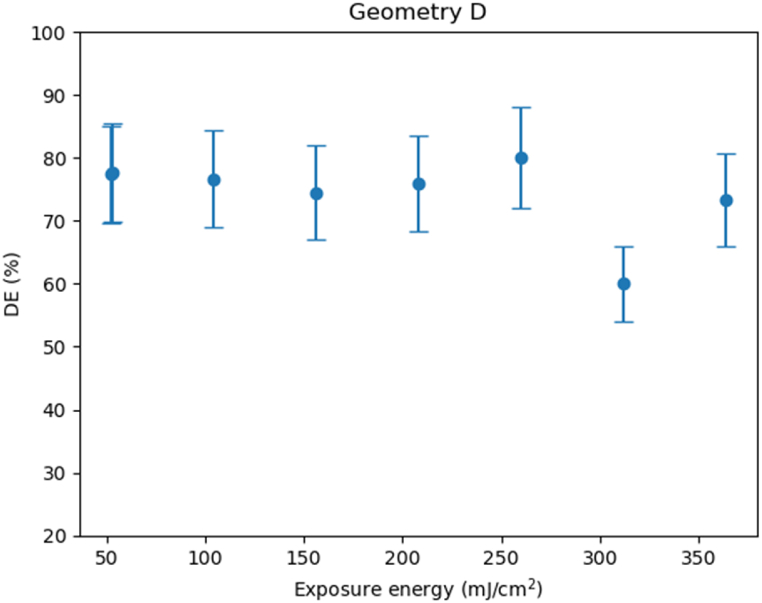
Diffraction efficiencies obtained using geometry D using the optimized intensities ratio for this geometry 0.6:1.5 mw/cm^2^.

Noteworthily, as shown in Fig. 9, this new geometry is interesting because the results of diffraction efficiency improve around 10% the ones obtained with geometry C.

### 3.4 Projection on screen, elimination of replicated images

**Fig. 10:**
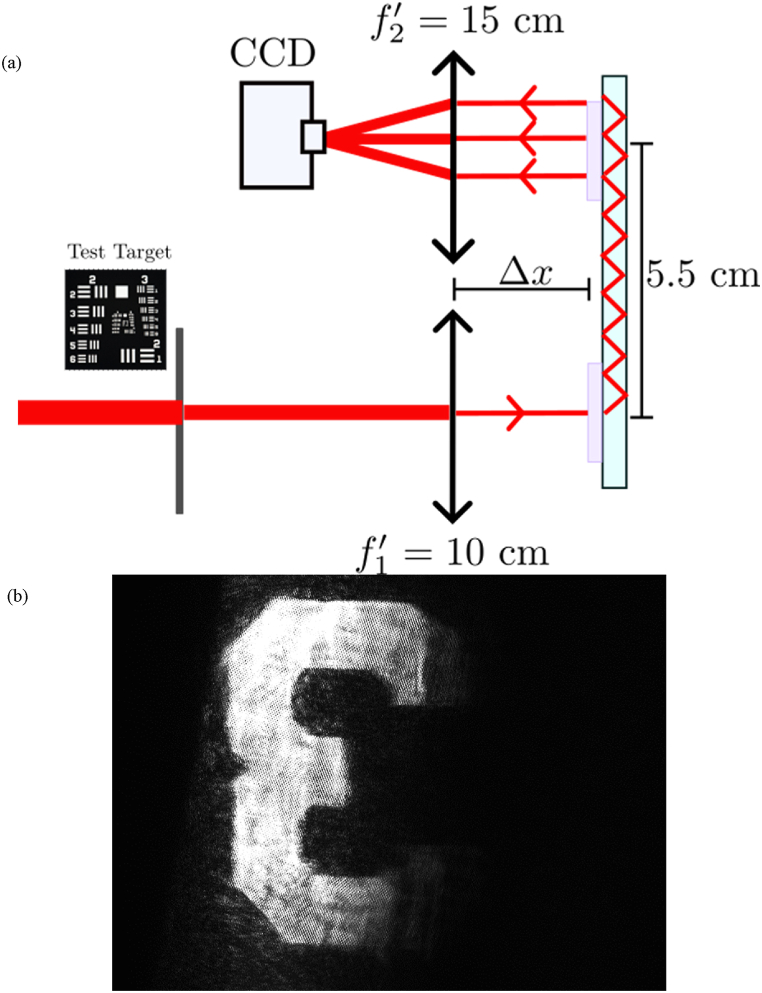
(a) Optic system added to the see-through display. (b) Image obtained with the system using 4-F system to converge the replicated images. Using normal incidence, Geometry A.

As we have shown in section 3.1, Fig. 6, one of the problems related to these displays is based on the multiple images extracted by the out-put coupler, regarding the total internal reflection propagation inside the waveguide. This phenomenon described in Ref. [20] is due to the diffraction efficiency of the out-coupler smaller than 100% and the Fresnel reflections. The Fresnel reflections are large for high tilted angles and refractive index variations from 1.505 to 1. Then we obtain the main image, where the light is more intense and many replicated images less bright. To solve this, for example in the case of projecting the image in a semitransparent screen, it is interesting to use a 4F system. The schematic performance of this experimental set-up is presented in Fig. 10 a.

One of the most significant advantages of this proposal is that by combining the focal lengths of the two lenses the magnification of the image can be modified. To check the system's behavior, we have used a negative test target R1DS1N provided by Thorlabs. The original size of the number 3 guided image is 6×4 mm and is magnified 1.5 times due to the optical system. The distance between the lenses and the glass Δx is measured to have a 4-F system considering the optical path of the light guided inside the glass, while the center of the in-coupler and out-coupler are distanced by 5.5 cm.

This system can just to initially demonstrate the imaging capabilities of the prepared device. It imitates what would be a real system of image formation of a see-through AR display in which we substitute the human eye (subjective system) for the projection screen (objective system).

To validate the effect of the optical system we have chosen the image of number 3 and a waveguide recorded with Geometry A, taking advantage of the Bragg angle being near normal incidence. As can be seen in Fig. 10 b, where the final image of the display is shown, using this common optical system all the replicated images converge in the main one, and the quality of the guided image is improved significantly. The extracted image is sharp and free of replications and the edges of the image are well contrasted and defined. This demonstration widens the landscape regarding the conjunction of an imaging system with our holographic waveguide.

## 4 Conclusions

We have optimized and studied a see-through system based on holographic in and out-couplers recorded in the commercial photopolymer Bayfol HX 200. We have demonstrated that we obtain better results using low recording intensities and the best performance is obtained with geometries C and D where the spatial frequency is around 1000 lines/mm. We also have studied the shrinkage of the photopolymer analyzing the first two Bragg’s angles, using the recording wavelength to calculate the recorded K vector, orders −1 and +1. We have demonstrated how the shrinkage helps to guide the light in our see-through geometry and open new possibilities to design recording geometries for in and out-couplers. Lastly, we have shown that by using a 4F system we can significantly improve the quality of the extracted image and modify the image magnification.

## Funding statement

Generalitat Valenciana (Spain) project PROMETEO/2021/006. Universidad de Alicante (UATALENTO18-10; ACIE-20-10). Generalitat Valenciana (Spain) (IDIFEDER/2021/014, cofunded by European Union through the FEDER Programme and “Ministerio de Ciencia e Innovación” of Spain (project PID2021-123124OB-I00).

## Author contribution statement

Cristian Neipp, Andrés Márquez: Conceived and designed the experiments. Sergi Gallego: Conceived and designed the experiments; Analyzed and interpreted the data; Wrote the paper. Joan Josep Sirvent-Verdú: Performed the experiments; Analyzed and interpreted the data; Wrote the paper. Juan Carlos Bravo: Performed the experiments. Jaume Colomina-Martínez: Analyzed and interpreted the data. Daniel Puerto: Contributed reagents, materials, analysis tools or data.

## Data availability statement

The data presented in this study are available on request from the corresponding author.

## Declaration of competing interest

The authors declare the following financial interests/personal relationships which may be considered as potential competing interests:

Sergi Gallego Rico reports financial support was provided by Government of Valencia. Sergi Gallego Rico reports a relationship with Spain Ministry of Science and Innovation that includes: funding grants.
